# Gene Polymorphisms in Chronic Periodontitis

**DOI:** 10.1155/2010/324719

**Published:** 2010-02-09

**Authors:** Marja L. Laine, Bruno G. Loos, W. Crielaard

**Affiliations:** ^1^Department of Oral Microbiology, Academic Center for Dentistry Amsterdam (ACTA), University of Amsterdam and Vrije University, 1081 BT Amsterdam, The Netherlands; ^2^Department of Periodontology, Academic Center for Dentistry Amsterdam (ACTA), University of Amsterdam and Vrije University, 1081 BT Amsterdam, The Netherlands

## Abstract

We aimed to conduct a review of the literature for gene polymorphisms associated with chronic periodontitis (CP) susceptibility. A comprehensive search of the literature in English was performed using the keywords: periodontitis, periodontal disease, combined with the words genes, mutation, or polymorphism. Candidate gene polymorphism studies with a case-control design and reported genotype frequencies in CP patients were searched and reviewed. There is growing evidence that polymorphisms in the *IL1, IL6, IL10*, vitamin D receptor, and *CD14* genes may be associated with CP in certain populations. However, carriage rates of the rare (*R*)-allele of any polymorphism varied considerably among studies and most of the studies appeared under-powered and did not correct for other risk factors. Larger cohorts, well-defined phenotypes, control for other risk factors, and analysis of multiple genes and polymorphisms within the same pathway are needed to get a more comprehensive insight into the contribution of gene polymorphisms in CP.

## 1. Introduction

Periodontitis like many other common diseases (e.g., Crohn's disease, cardiovascular diseases, diabetes) is considered to be a complex multifactorial disease. Typical for complex human diseases is that they mostly have a relatively mild phenotype and are slowly progressing and chronic in nature. Furthermore, these diseases are of relative late of onset (i.e., postjuvenile or adult onset) and are relatively common. The phenotype of the complex diseases is determined by both genetic and the environmental factors that affect the individual. Although pathogenic bacteria and various other environmental factors (e.g., smoking and stress) [1] are involved in pathogenesis of periodontitis, also genetic factors are evidenced in the aetiology of periodontitis [2, 3]. 

Understanding of the interplay between the host and oral bacteria is essential to the understanding of the pathogenesis of periodontal disease. Periodontopathic bacteria initiate and repeatedly attack the host, which subsequently reacts with immune response and may slowly destruct by the action of the inflammatory process itself. However, the presence of pathogenic subgingival bacteria alone does not result in periodontal destruction in most cases. Therefore, although bacteria are essential for the initiation of periodontitis, the amount of plaque and the species of bacteria does not necessarily correlate with disease severity [4]. Each person may have an individual dose dependend response to the bacterial challenge that determines his/her susceptibility to periodontitis. Most individuals are resistant to the disease and will not develop periodontitis.

There are a large number of scientific papers searching for the role of genes and their variants (polymorphisms) in host responses in periodontitis, and in the progression of the disease. The genetic polymorphisms may in some situations cause a change in the protein or its expression possibly resulting in alterations in innate and adaptive immunity and may thus be deterministic in disease outcome. Genetic polymorphisms may also be protective for a disease. The pathophysiology of periodontitis, as of other complex diseases, is characterized by various biological pathways leading to the same clinical phenomena. Multiple genes and their polymorphisms may all have a small overall contribution and relative risk to disease susceptibility and severity. Complex diseases are typically polygenic [23]. The disease genes in complex diseases are therefore considered modifying disease genes [24]. It is important to realize that the number and type of modifying disease genes for the same disease may not be same in different ethnic populations. In the present review we explore and summarize literature up to April 2009 on putative genetic risk factors in chronic periodontitis (CP) susceptibility.

## 2. The Role of Genetics in Chronic Periodontitis

Evidence for the role of genetic component in chronic (adult) periodontitis has been conducted from twin and family studies. The twin model is probably the most powerful method to study genetic aspects of any disease, including periodontal disease. Michalowicz et al. evaluated the periodontal conditions (attachment loss, pocket depth, gingival index, and plaque index) of 110 adult twins with a mean age of 40 years ranging from 16 to 70 years [3]. The results indicate that between 38% and 82% of the population variance for these measures may be attributed to genetic factors. In a study on 117 adult twin pairs [2] the analysis included the evaluation of the environmental factors like smoking and utilization of dental services. The results showed that chronic (adult) periodontitis was estimated to have approximately 50% heritability, which was unaltered following adjustments for behavioral variables including smoking. In contrast, there was no evidence of heritability for gingivitis after behavioral covariates such as utilization dental care and smoking were incorporated in the analysis. 

Velden et al. [33] studied with a family study design the effect of sibling relationship on the periodontal condition in a group of young Indonesians deprived from regular dental care. The results of the analysis suggest that also in less severe forms of periodontitis there may be a genetic background for the disease. Also in a Dutch population epidemiological studies have suggested that chronic (adult) periodontitis aggregates in families [34].

From both the twin and family studies it can be concluded that the basis for familial aggregation of periodontitis appears not bacterial/environmental/behavioral in nature; rather, genetics seem to form the basis for the familial aggregation of periodontitis.

## 3. Strategy of the Recovery of Published Data

A comprehensive literature search on the PubMed database up to April 2009 was conducted using the keywords: Periodontitis, Periodontal disease, in combination with the words Genes, Mutation, or Polymorphism. The studies selected for the review (1) were written in English, (2) had a case-control design including patients with chronic (CP) or adult (AP) periodontitis, and (3) reported genotype distribution. 

In the preparation of this review we encountered various nomenclatures for the diagnosis of cases and controls, and during the years, the nomenclature for the diagnosis of the various forms of periodontitis has changed. In this paper we have used the diagnosis of periodontitis from the original manuscripts as much as possible. We focussed on the role of genetic polymorphisms (mainly single nucleotide polymorphisms [SNPs]) in chronic periodontitis (CP) susceptibility.

In the present review, the most common variant of the polymorphic locus in the study population is nominated as a *normal* variant (*N*-allele). Thus, if a locus is, for example, bi-allelic, the less frequent allele is designated as a *rare *variant (*R*-allele) but must occur with a frequency of >1% in the population. We present in the tables the frequency of the carriage rate of the *R*-allele (frequency of *N/R* and *R/R* genotypes) among cases and controls. In addition we present in the tables whether or not the authors of the cited papers have reported statistically significant differences between cases and controls for a given *R*-allele. 

Most genetic research in periodontitis has focused on gene polymorphisms that play a role in the immune system, tissue destructive processes, or metabolism mechanisms. Tables 1–12 present the candidate gene polymorphisms investigated in relation to CP susceptibility. In this review a polymorphism was considered to be associated with CP susceptibility if a polymorphism has been investigated in several studies and the association has been replicated at least once. Below we discuss the findings of various epidemiological studies that were undertaken to further understand the roles of gene polymorphisms in susceptibility to chronic periodontitis.

## 4. Candidate Genes in Relation to Chronic Periodontitis (CP)

### 4.1. Polymorphisms in the IL1 Gene Cluster

Interleukin-1 (IL-1) is a potent proinflammatory mediator that is mainly released by monocytes, macrophages, and dendritic cells. Levels of IL-1*α* and IL-1*β*, (proinflammatory cytokines) and IL-1/IL-receptor antagonist (RA, antiinflammatory cytokine) ratio have been found to be increased in diseased periodontal tissues and gingival grevicular fluid [53, 54]. The genes encoding for the proteins IL-1*α*, IL-1*β* , and IL-1RA are located in close proximity in the *IL1* gene cluster on chromosome 2q13–q21. The *IL1A *-889 and *IL1B* +3953 *R*-alleles have been shown to increase and the *IL1RN* VNTR* R*-alleles to decrease gene transcription or the protein production levels [17, 55, 56] resulting in the *R*-allele carrier individuals in a more pronounced IL-1 pro-inflammatory response.

The *IL1* genotypes appear to be the most studied genetic polymorphisms in CP (Tables 1–4). Kornman et al. [36] reported on a composite genotype, composed of the *IL1A *-889 and *IL1B* +3953 polymorphisms both carrying an *R*-allele, in relation to periodontitis. To date, the following *IL1* genetic polymorphisms have been studied in association with chronic periodontitis: *IL1A *-889 (in linkage disequilibrium with +4845), *IL-1B *-511 (in linkage disequilibrium with -31), *IL1B* +3954 (also mentioned in the literature as +3953), and *IL1RN* VNTR (in linkage disequilibrium with +2018). 

Results of case-control studies in Caucasians and non-Caucasians are presented in Tables 1–4. From the tables it becomes clear that among the different studies even exclusively within Caucasian subjects, considerable variation is seen for the carriage rates of the *IL1 R-*alleles. For example, for the polymorphic *IL1A *-889 (+4845) (Table 1), the carriage rate for the *R-*allele varies from 43% to 90% in patients and from 35% to 79% in controls. The carriage rate of the *IL1A *-889 (+4845) *R-*allele in Asian populations appears low (8%–23%) [18, 20] in comparison to other populations. The latter finding demonstrates an important issue, that is, the carriage rate of genetic polymorphisms may vary among different ethnic populations. Therefore, possible positive associations between a genetic polymorphism and disease within one population may not necessarily be extrapolated to other populations. Only two studies [14, 22] have reported on an association between the carriage rates of the *IL1A *-889* R-*alleles and CP as a single genetic risk factor. 

The SNP *IL1B* +3954 (+3953) was initially proposed as risk factor for periodontitis among Caucasians (Table 2). Nevertheless there are conflicting results. Galbraith et al. [25] found an association between the *R-*allele and periodontitis, and Gore et al. [5] observed an association with the severity of periodontal destruction. Also Lopez et al. [11], Moreira et al. [30], and Wagner et al. [14] have associated the *IL1B* +3954* R-*allele with CP. However, Rogers et al. [8] did not find the association for the *R*-allele but for the *N*-allele in CP. Among Asian subjects, the carriage rate of the *IL1B* +3954 (+3953) *R-*allele is importantly lower (≤10%) [18, 20, 29] than that in Caucasian populations (13%–74%) (Table 2). Struch et al. [15] have performed a large scale study on the *IL1B* +3954 polymorphism in a Caucasian population: in a group of 893 CP patients and 493 controls carriage rates for the *R*-allele were 44% and 39%, respectively, which was not significant (*P* = .07).

Four studies have reported carriage rates for the *IL1B *-511 (-31) *R-*allele, and to date this genetic polymorphism has not been associated with CP (Table 3). The carriage of the *R-*allele was higher among Japanese (67%) than among Caucasians (43%–59%) [5, 10, 16, 29].

Few studies have investigated polymorphisms in the *IL1RN* gene, encoding the IL-1RA (Table 3) and again conflicting results are reported. The *R-*allele carriage is associated as a single genetic risk factor with CP (45% versus 7% in controls) in Turkish Caucasians [35]. In combination with *IL1A *-889 and *IL1B *+3954, the *IL1RN R*-allele was reported to have a relationship with periodontitis susceptibility [6].

Kornman et al. [36] reported that the combined presence of the *R-*allele of the *IL1A* *!*gene at nucleotide position –889 and the *R-*allele of the *IL1Ḅ* gene at nucleotide position +3954 (+3953) was associated with severity of periodontitis in nonsmoking Caucasian patients. This combined carriage rate of the *R-*alleles was designated the *IL1* composite genotype [36]. Since that time a considerable number of studies investigating the *IL1* composite genotype have been published in Caucasians and non-Caucasians (Table 4). Studies on Caucasian populations have shown prevalence from 10% to 46% for the composite genotype, whereas among Asian populations [18, 20, 40] prevalence of the *IL1* composite genotype was very low (≤3%). 

After the initial results of Kornman et al. [36], many case-control studies have investigated the *IL1* composite genotype as a putative risk factor for CP susceptibility, mostly in Caucasian populations (Table 4). Two studies have observed an association between the *IL1* composite genotype and periodontitis susceptibility in Caucasians [11, 37] and one study in non-Caucasians [41]. Meisel et al. [39] observed the *IL1* composite genotype to be associated with periodontitis in Caucasian but only in smokers. However, all other studies have failed to replicate this association (Table 4). Nevertheless, it has also been reported that patients with the *IL1* composite genotype more often harbored putative periodontal pathogens and have increased counts of these pathogens [147]. Interestingly, Laine et al. [6] reported increased frequency of the *R*-alleles of the *IL1A, IL1B, *and* IL1RN* genes in non-smoking patients in whom the periodontal pathogens *Porphyromonas gingivalis* and *Aggregatibacter actinomycetemcomitans* could not be detected. These latter results suggest that *IL1* gene polymorphisms may play a role in the absence of other (putative) risk factors. 

Taken altogether, the *IL1* gene cluster polymorphisms cannot be considered as risk factors for CP susceptibility for the worldwide population. However, for Caucasian CP patients the *IL1* composite genotype and/or *IL1B* +3953 genotype may be genetic risk factors. Results of the meta-analysis of Nikolopoulos et al. [148] support also an association between CP and *IL1A *-889 and *IL1B* +3953 *R*-allele carriage as well separately as in composite genotype in Caucasians.

### 4.2. Polymorphisms in the TNFA Gene

Tumor necrosis factor (TNF) is a proinflammatory cytokine that possesses a wide range of immunoregulatory functions. TNF is produced by monocytes, macrophages, and lymphocytes and has the potential to stimulate the production of secondary mediators, including chemokines or cyclooxygenase products, which consequently amplify the degree of inflammation. The *TNFA* gene is located on chromosome 6p21.3 within the Major Histocompatibility Complex gene cluster. Several case-control studies in both Caucasians and non-Caucasians have investigated genetic polymorphisms in the *TNFA* gene as putative risk factors for periodontitis. SNPs in the gene encoding *TNFA* are mainly studied in the promoter region at positions -1031, -863, -857, -376, -308, and -238 but also in the coding region in the first intron at position +489. The results of these studies are summarized in Table 5. 

The differences in the carriage rate of the *R-*alleles between Japanese and other populations are apparent; at position -308 the *R*-allele carriage rates for Japanese subjects were only 2%-3% (Table 5) [29] and for other populations 18%–44% [10, 12, 13, 25, 43–47]. For the *TNFA *-238 the frequencies of *R*-alleles were comparable between different ethnic populations (<15%) (Table 5). For the other *TNFA* polymorphisms only single studies have been reported, and positive associations with CP have been found for the -1031, -863, and -857 loci [29]. 

To date there is very limited data to support associations between any of the reported *TNFA* gene variations and CP susceptibility. Although the SNP's *TNFA *-1031, -857, and -863 showed an association with CP in Japanese, these findings have not been replicated [29].

### 4.3. Polymorphisms in the IL4 and IL4RA Genes

Interleukine-4 (IL-4) is a pleiotropic cytokine, which is produced by the T helper 2 cell subpopulation and can rescue B lymphocytes from apoptosis and enhance their survival, thus promoting B-lymphocyte mediated immunity. IL-4 also downregulates macrophage function [149]. The gene for *IL4* has been located on chromosome 5q31.1.

Gene polymorphisms studied in the *IL4* gene are summarized in Table 6. An* IL4* -590 promoter polymorphism and a 70-bp VNTR polymorphism are the most studied polymorphisms of *IL4*. Case–control studies have not shown any relationship between the *IL4* gene polymorphims and susceptibility to CP in several different populations. However, a haplotype of *IL4 *polymorphisms (carriers of *R-*alleles in all three SNPs studied) has been associated with CP (17.0% in cases versus 11.0% in controls; OR 1.85) [49]. No association was found for the *IL4RA* polymorphims in a study on Caucasians [46].

### 4.4. Polymorphisms in the IL6 and IL6R Genes

Multiple roles have been identified for interleukine-6 (IL-6). It is released by different cell types and its secretion levels are determined by the cell type and the nature of the stimulus [150, 151]. The *IL6* gene was demonstrated to be localized on chromosome 7p21. *IL6 *polymorphisms affect the serum levels of circulating interleukin-6. The -174 was found to influence *IL-6* expression and production. The -174 *R-*allele carrier individuals have decreased plasma levels of IL-6 and present lower *IL6* gene transcriptional activity when compared with *N/N* individuals [152]. Therefore a genetically determined low IL-6 response (the -174 *R-*allele carriers) may hamper individual's defense against periodontal pathogens.

The carriage rates of the *IL6 *-174 *R-*allele varied in Caucasian populations from 44% to 54%, in Brazilian populations from 37% to 67%, and remarkably, the -174 as well as -190 and -597 loci were nonpolymorphic in a Japanese population (Table 7). Three out of six studies in Caucasian and one out of two studies in Brazilian populations have correlated the *IL6 *-174 G>C polymorphism with susceptibility to CP. With regard to the other *IL6* gene polymorphisms, the Czech study [57] suggested that the -572 polymorphism may be a protective factor to CP. Furthermore, for the other *IL6* polymorphisms only single studies have been reported. 

We conclude that the *IL6 *-174 polymorphism may be associated with CP susceptibility. However, a meta-analysis of the *IL6 *-174 polymorphisms did not show any association for this polymorphism with CP [148].

### 4.5. Polymorphisms in the IL10 Gene

Interleukine-10 (IL-10) is considered an antiinflammatory cytokine, downregulating the proinflammatory immune response of the monocytes and macrophages. However, the B lymphocyte stimulatory effect may also stimulate the production of autoantibodies [153]. As a matter of fact, auto-antibodies may play a role in periodontitis [154–156]. IL-10 is produced by monocytes, macrophages, and T cells and plays a role in the regulation of proinflammatory cytokines such as IL-1 and TNF-*α*.

The gene encoding for IL-10 is mapped on chromosome 1q31-q32, in a cluster with closely related interleukin genes, including *IL-19, IL-20,* and *IL-24*. Several promoter polymorphisms have been described in the *IL10* gene: -1087 (-1082), -819 (-824), -627, -592 (-597), and -590 (Table 8). The IL-10 -1082, -819, and -592 polymorphisms show strong linkage disequilibrium and form two common haplotypes. The haplotypes may be determined on basis of the *IL10 *-592 polymorphism [69]. The *R*-allele of the -592 polymorphism has been associated with decreased synthesis of IL-10 in vitro and in vivo [157, 158] and may lead to altered synthesis of IL-10 in response to inflammatory stimuli [69]. IL-10 has a protective role towards periodontal tissue destruction, inhibiting both matrix metalloprteinases (MMP) and receptor activator for nuclear factor-*κ*B (RANK) systems [159, 160]. Therefore the *IL10 *-*592 R*-allele carriers may be less protected against bacterial challenge. 

Table 8 summarizes the case-control studies investigating genetic polymorphisms in the *IL10* gene in association with CP susceptibility. The carriage rates of the *IL10* -1087 *R*-allele vary between 44% and 81% in Caucasians. The -1087 locus has not been associated with CP susceptibility in most of Caucasian populations. However, the -1087 *N*-allele was associated with CP in Swedish Caucasians [65]. 

The *IL10 *-819 polymorphism has been correlated with CP in Brazilians but not in other populations [67]. Until now all three studies on the *IL10 *-592 polymorphism have found a higher *R*-allele carriage rate in CP patients [67–69]. The *IL10 *-592 *R*-allele carriage rates varied in different populations between 68% and 75% in CP patient and between 41% and 51% in controls.

One study on Japanese CP patients (*N* = 34) and controls (*N* = 52) analyzed haplotypes consisting of the *IL10 *-1087, -819, and -592 gene polymorphisms [161]. Only haplotype frequencies were reported and no separate genotype frequencies were presented. No significant differences for the carriage rates of the haplotypes of the *IL10* gene were found between patients and controls. Striking was the complete absence of the *N*-allele carriage at position -1087 among the Japanese, in contrast to Caucasians (Table 8), where the -1087 *N*-allele is the most occurring variant [65, 161].

For conclusion, *IL10 *-592 *R*-allele carriage rates have been associated with CP susceptibility and the results have been replicated [67–69]. Therefore we conclude that the *IL10 *-592 polymorphism may be a genetic marker for CP susceptibility.

### 4.6. Polymorphisms in the Fc*γ*R Gene

Leukocyte receptors for the constant (or Fc-) part of immunoglobulin (FcR) link cellular and humoral parts of the immune system, which are considered essential for the host defense against bacteria.

Fc*γ*Rs are found on a wide variety of immune cells in the periodontal tissues [162]. Fc*γ*Rs are likely to play a role in the pathogenesis of periodontitis [163]. Microorganisms and bacterial antigens, opsonized with antibody, can be phagocytosed via Fc*γ*R on neutrophils or internalized via Fc*γ*R by a variety of antigen presenting cells, including monocytes, macrophages, and B cells. T cells and natural killer cells may become activated, when IgG-opsonized bacteria are bound to these cells via Fc*γ*R; a variety of cytokines and chemokines may also be released [164]. 

The Fc*γ*R genes are found on chromosome 1 and encode 3 main receptor classes: Fc*γ*RI (CD64), Fc*γ*RII (CD32), and Fc*γ*RIII (CD16). These classes are further subdivided into subclasses: Fc*γ*RIa and b, Fc*γ*RIIa, b, and c, and Fc*γ*RIIIa and b. Structural and functional differences in Fc*γ*RIIa, IIIa, and b have been described [164, 165]. 

The studies that have investigated the *Fc*γ*RIIa, Fc*γ*RIIIa, *and *Fc*γ*RIIIb* polymorphisms in relation to periodontitis are summarized in Table 9. Several studies have investigated the *Fc*γ*RIIa* polymorphisms in relation to CP. In Caucasians, the carriage rate of the *Fc*γ*RIIa R-*allele is relatively high: 63%–76% [70–73] and in Asian populations the carriage rate is lower: 36%–62% (Table 9). In general, the *Fc*γ*RIIa* polymorphisms are not associated with CP. However, Yamamota et al. [72] observed a decreased prevalence of the Fc**γ**RIIa *R-*allele among Caucasian CP patients and controls in a large case-control study. Homozygosity for the *N*-allele was significantly more prevalent in smoking CP patients [72]. 

A lower *R-*allele carriage rate of the *Fc*γ*RIIIa* gene is seen in Japanese in comparison to the Caucasians. In a Japanese population it was found that the *Fc*γ*RIIIa R-*allele was over-represented in patients with periodontal disease recurrence [78]. In contrast, another Japanese study showed that the *Fc*γ*RIIIa N*-allele was overrepresented in patients with severe periodontitis versus subjects with moderate disease [76]. But none of the studies have associated the *Fc*γ*RIIIa* polymorphisms with CP susceptibility. It is apparent that there are conflicting results and comparisons between the different studies are difficult as the prevalences of *Fc*γ*R* genotypes differ among subjects of different ethnic background.

The carriage rate of the *Fc*γ*RIIIb R*-allele in Caucasians was relatively high (>75%) and in Asians some what lower (55%–74%). In Caucasians no associations have been found between the *Fc*γ*RIIIb R*-allele carriage and CP susceptibility. However, in one Japanese study the *R-*allele carriage has been associated with CP susceptibility [79]. Two studies of Kobayashi et al. [74, 76] have shown an association with CP disease recurrence and severity in combination with *Fc*γ*RIIIa N*-allele.

Initially, polymorphisms in the *Fc*γ*R* genes were suggested to play a role in periodontitis [166]; however in the present review on the susceptibility to CP, only one study out of ten found CP to be associated with *Fc*γ*R*IIa polymorphism in smokers [72], and one out of nine studies with *Fc*γ*R*IIIb [79]. Therefore we conclude that the reported *Fc*γ*R* gene polymorphisms are not associated with CP susceptibility. However, to date no large-scale epidemiological investigations are available, and subsequently no clear and convincing data is presented to assign the *Fc*γ*R* gene polymorphisms as risk factors for CP.

### 4.7. Polymorphisms in the VDR Gene

Vitamin D plays a role in bone metabolism. Since alveolar bone resoption is a major characteristic of periodontal disease, it is plausible that mediators of bone metabolism like the vitamin D receptor (*VDR*) and its' genetic polymorphisms play a role in CP susceptibility. In addition to mediating bone homeostasis, vitamin D and its receptor play a role in phagocytosis by monocytes and affect monocyte differentiation [167].

The human *VDR* gene is localized on chromosome 12q12–q14. Genetic polymorphisms in the *VDR* gene have also been associated with infectious diseases, in particular tuberculosis [168, 169]. The mechanisms by which *VDR *gene polymorphisms may influence CP susceptibility have not been clarified yet. The *Taq1, Bsm1, *and *Apa1 *polymorphisms do not change the translated protein whereas the *Fok1* polymorphism may be functional creating an additional start codon (ACG to ATG) [170]. 

Several studies have identified *VDR* polymorphisms in relation to CP at RFLP positions *Taq1, Bsm1, Fok1, *and *Apa1 *(Table 10) [10, 80–86]. Most of the studies on the SNPs of the *VDR* gene have found associations with CP, however not always unconditionally (Table 10). 

The carriage rates of the *VDR Taq1 R*-allele range between 42% and 78% across different ethnic populations, except in Asian populations where lower rates (4%–23%) have been reported (Table 10). Not the *Taq1 R*-allele but the *N*-allele has been associated with CP susceptibility in several studies (Table 10). Another *VDR* polymorphism (*Bsm1*) showed no association with CP as a single SNP but in different haplotype combinations with the other *VDR* polymorphisms [84–86]. 

The *VDR* gene is an interesting candidate gene for its association with periodontitis, because it affects both bone metabolism and immune functions. The *VDR Taq1* SNP may be associated with CP susceptibility as a single polymorphism or in combination with other *VDR* gene polymorphisms.

### 4.8. Polymorphisms in the Pattern Recognition Receptor Genes

The innate immune system recognizes pathogen-associated molecular patterns (PAMPs) that are expressed on microorganisms, but not on host cells. Extra- and intracellular receptors like CD14, CARD15, and Toll-like receptors (TLRs) recognize PAMPs of Gram-positive and Gram-negative bacteria and mediate the production of cytokines necessary for further development of effective immunity. Both TLR2 and TLR4 use CD14 as a coreceptor.

#### 4.8.1. Polymorphisms in the CD14 Gene

The gene for CD14 is located on chromosome 5q21–q23. The *CD14 *-260 (-159) promotor polymorphism is located upstream from the major transcriptional site, affecting the transcriptional activity and CD14 density [171]. Individuals homozygous for the *R-*allele have increased serum levels of soluble (s) CD14 and an increased density of CD14 in monocytes [171]. The *CD14 *-260 *R-*allele has previously been associated with increased risk of myocardial infarction [171] and Crohn's disease [172]. Given that the *CD14* –260 *N*-allele leads to a reduced expression of the CD14 receptor it is assumed that individuals carrying the *N*-allele may be more susceptible to CP since they are less protected by the CD14 receptor [173].

Carriage rate of the *CD14* –260 *R-*allele varies in different ethinic populations from 47% to 82%. Eight studies have investigated the *CD14 *-260 polymorphism in Caucasian CP subjects (Table 11), but the results are conflicting. Two studies found an association with the *N*-allele and another study with the *R*-allele whereas five studies did not find any association with the CP susceptibility [87, 93]. 

Results for another polymorphism (position -1359) in the *CD14* gene have also been reported [87, 90]; no association with CP susceptibility was found. However a higher frequency of the *N*-allele and the *N/N* genotype of the *CD14 *-1359 polymorphism was found in patients with severe periodontal disease than in patients with moderate periodontitis (Table 11) [87]. 

We conclude that the *CD14 *-260 polymorphism may be associated with CP susceptibility.

#### 4.8.2. Polymorphisms in the TLR2 and TLR4 Genes

*TLR 2* and *TLR4* genes map on chromosome 4q32 and 9q32-q33, respectively. *TLR2* Arg677Trp and Arg753Gln gene polymorphisms have been reported to change the ability of TLR2 to mediate a response to bacterial components [174]. Two common cosegregating missense polymorphisms of *TLR4, *Asp299Gly and Thr399Ile, affect the extracellular domain of the TLR4 protein, leading to an attenuated efficacy of LPS signalling and a reduced capacity to elicit inflammation [175]. The *TLR4 *Asp299Gly gene polymorphism has been correlated with sepsis and infections caused by Gram-negative bacteria [176]. The above named *TLR* polymorphisms have been studied by several groups in association with periodontitis (Table 11) [10, 13, 89–91, 94–97, 177]. However, in spite of the perceived importance of these functional *TLR* polymorphisms, no relation with CP has been observed. Nine SNPs in the *TLR 2* and *TLR4* genes have been studied by Fukusaki et al. [95] in a Japanese population, and *TLR4* +3725 polymorphism was found to be associated with CP.

Interestingly, the *TLR2* 677 loci was not polymorphic in Caucasian and Japanese populations [94, 95, 177], but the heterozygotic genotype was found in 100% of the Han Chinese [96]. The *TLR2 *753 and the *TLR4* polymorphisms were not or in very low percentage polymorphic in Asian populations. In Caucasian populations the *TLR4* 299 and 399 carriage rates of the *R*-allele ranged between 4% and 25% (Table 11).

Although the pattern recognition receptor genes seem good candidates for their association with periodontitis, investigations have not yielded any strong indications that they might be associated with CP susceptibility.

### 4.9. Polymorphisms in Miscellaneous Genes

Miscellaneous candidate gene polymorphisms that have been studied in relation to CP are listed in Table 12. These are not discussed in detail as the other candidate genes above, since mainly negative results and/or too few studies are published for a meaningful analysis. However, Table 12 illustrates the variety of candidate genes and the difficulty in interpreting results; if positive results are reported, these are often in subgroups or conditionally.

## 5. Discussion and Conclusions

Case-control association study design is considered a powerful method in detecting high frequently occurring, small-effect gene polymorphisms. However, this study design is susceptible to a variety of potential methodological flaws. An important concern is selection of case and control subjects because it has a great impact on study outcome. To be able to detect genetic polymorphisms playing a role in disease predisposition, strict phenotype classification should be employed during the selection procedure of the study subjects. Importantly, the clinical symptoms like deepening of the periodontal pocket, loss of attachment, and alveolar bone loss are same in different forms of periodontal diseases. Also definition of control subjects may vary in different studies. Some reports characterize their control subjects as healthy, while others describe their control subjects as gingivitis patients or population controls. Inaccuracy in disease classification of CP makes the case-control studies and replication of the studies difficult. 

Another possible bias in case-control studies is the diversity of ethnic background of study cohorts. Since genotype and allele frequencies may differ between different ethnic populations [178], case and control subjects should be selected on the basis of the same ethnic background. A genetic risk factor for disease susceptibility in one population may not be a risk factor in the other population.

From the current review, it became clear that a fairly large number of studies on CP susceptibility are limited by their sample size and power. Subsequently, no gene polymorphism has, as yet, been definitely shown to be a risk factor for CP susceptibility. Small sample size studies are greatly underpowered, since most associations refer to small odds ratio's (range 1.1–1.5) and greatly contribute to the risk for false positive or negative results [179]. For instance, approximately 2000 cases and 2000 controls would be required to provide 80% power to detect an odds ratio of 1.5 at a *R*-allele frequency of 0.1 and at an appropriate level of significance [180]. However many disease susceptibility polymorphisms will confer an odds ratio less than 1.5, requiring larger patient cohorts. Sufficient number of cases and controls must be recruited in order to minimize the risk of identifying false positive associations that are due to chance alone or, conversely, of failing to detect a true association between a polymorphism and a disease (false negatives). 

Typical for the multifactorial and polygenic complex diseases is that each genetic polymorphism has generally only a modest effect, and that the interaction of genes and their polymorphisms with each other (gene-gene interaction) and with environmental factors (gene-environment interaction) potentially has influence on the observed phenotype. Therefore, multivariate analyses should be included to generate odds ratios taking into account next to age and gender-established risk factors like smoking, microbial factors, and eventually interaction with other gene polymorphisms. 

In case-control studies selection of candidate genes and their polymorphisms is based on *a priori* knowledge of disease pathogenesis and phenotypes. Consequently, one of the greatest challenges in candidate gene studies remains the intelligent selection of candidate genes and their polymorphisms. However the amount of knowledge, to date, is enormous and effective computer-based methods may be helpful for deciding *a priori* which genes, polymorphisms, and combinations (haplotypes) have the greatest chance of influencing disease susceptibility [181, 182]. Most genetic research on CP susceptibility has focused sofar on gene polymorphisms that play a role in the recognition and clearance of bacteria by the immune system, tissue destructive processes, or metabolic mechanisms. 

Meta-analyses may be a helpful approach in rationalizing the results from several small and conflicting studies. Once a considerable amount of studies are available, meta-analyses may be performed to pool data from different studies and determine allele frequencies in the different populations. However, meta-analyses may still have inherent problems such as including individual studies that employ widely different phenotype criteria, and publication bias. Previously, it has been demonstrated that molecular genetic research is sensitive to “negative” publication bias [183]. Evidently, further studies on gene polymorphisms in CP susceptibility are needed employing large amounts of individuals. Definite conclusions can be drawn on basis of multiple, large-scale studies. Consortia and collaborative studies may help to defeat the limitations of the individual studies.

In conclusion, research on genetic polymorphisms in the recent years has had limited success in unravelling significant and reproducible genetic factors for susceptibility to CP. Taken together the data published so far on gene polymorphisms in CP, we conclude that at this point there is a relatively large variation among the various studies for the *R*-allele carriage rates, even if the study populations are of the same ethnic background. Nevertheless, some evidence is emerging that polymorphisms in the *IL1, IL6, IL10, VDR,* and *CD14 *genes may be associated with CP susceptibility in certain populations. Future studies should apply more strict disease classification, larger study cohorts, adjust for relevant risk factors in CP, and include analysis of multiple genes and polymorphisms. Novel statistical methods may allow a better assessment of multiple genes and polymorphisms within the same pathway and interactions with environmental factors. The possibility to include data from multiple genes and polymorphisms or haplotypes and environmental data, and to model their interactions, will give us a better assessment of CP and its pathophysiology. 

## Figures and Tables

**Table 1:** *IL1A *-889 (+4845) C>T gene polymorphisms and carriage rate of the *Rare* (*R*)-allele in case-control studies and association with susceptibility to chronic periodontitis.

	Patients		Controls			
Ethnicity	*n*	*R*-allele	*N*	*R*-allele	Associated	Reference
of subjects		carriage		carriage	with periodontitis	
Caucasian	32^2^	43%	32	38%	−	Gore et al.1998 [5]
Caucasian	105^2^	64%	53	60%	− (+^4^)	Laine et al. 2001 [6]
Caucasian	61	43%	800	50%	−	Thomson et al. 2001 [7]
Caucasian	84^1^	48%	60	45%	−	Rogers et al. 2002 [8]
Caucasian	45	53%	110	43%	−	Sakellari et al. 2003 [9]
Caucasian	57	72%	100	56%	−	Brett et al. 2005 [10]
Caucasian	330^3^	44%	101	35%	−	Lopez et al. 2005 [11]
Caucasian	56	54%	90	49%	−	Sakellari et al. 2006 [12]
Caucasian	51	55%	178	43%	−	Tervonen et al. 2007 [13]
Caucasian	97	90%	97	79%	+	Wagner et al. 2007 [14]
Caucasian	893^2^	54%	493	49%	−	Struch et al. 2008 [15]
Caucasian	51^1^	71%	168	60%	−	Geismar et al. 2008 [16]
Mixed^1^	83	69%	37	52%	−	Shirodaria et al. 2000 [17]
Asian (Thai)	54	8%	43	23%	−	Anusaksathien et al. 2003 [18]
Japanese	58^3^	14%	44	16%	−	Kobayashi et al. 2007 [19]
Japanese	100^3^	20%	100	16%	−	Kobayashi et al. 2007 [20]
Brazilian	29	14%	17	23%	−	Gonçalves et al. 2006 [21]
Brazilian	67	60%	41	41%	+	Moreira et al. 2007 [22]

nr = not reported. − = association not found. +  = association found.

^1^63% Caucasian; 22% Asian; 15% Afro-Caribean.

^2^Cases diagnosed as adult periodontitis.

^3^Cases diagnosed as mixed periodontitis status.

^4^An association with periodontitis was found for combined genotype: carriage of *R*-allele for *IL1A *-889, *IL-1B* +3954, and *IL1RN *in a subgroup of patients being nonsmokers, and at the same time culture negative for *P. gingivalis *and* A. actinomycetemcomitans.*

**Table 2:** *IL1B* +3954 (+3953) C>T gene polymorphisms and carriage rate of the *Rare* (*R*)-allele in case-control studies and association with susceptibility to chronic periodontitis.

	Patients		Controls			
Ethnicity	*n*	*R*-allele	*n*	*R*-allele	Associated	Reference
of subjects		carriage		carriage	with periodontitis	
Caucasian	32^1^	43%	32	38%	−	Gore et al. 1998 [5]
Caucasian	40^1^	50%	45	27%	+	Galbraith et al. 1999 [25]
Caucasian	105^1^	49%	53	45%	− (+^3^)	Laine et al. 2001 [6]
Caucasian	61	34%	800	41%	−	Thomson et al. 2001 [7]
Caucasian	84^1^	35%	60	40%	+ ^4^	Rogers et al. 2002 [8]
Caucasian	28^1^	46%	33	48%	−	Gonzales et al. 2003 [26]
Caucasian	45	49%	110	50%	−	Sakellari et al. 2003 [9]
Caucasian	57	42%	100	41%	−	Brett et al. 2005 [10]
Caucasian	330^2^	30%	101	13%	+	Lopez et al. 2005 [11]
Caucasian	32	34%	52	40%	−	Droździk et al. 2006 [27]
Caucasian	13	33%	13	33%	−	Gustafsson et al. 2006 [28]
Caucasian	56	41%	90	44%	−	Sakellari et al. 2006 [12]
Caucasian	51	49%	178	44%	−	Tervonen et al. 2007 [13]
Caucasian	97	74%	97	43%	+	Wagner et al. 2007 [14]
Caucasian	51^1^	57%	168	43%	−	Geismar et al. 2008 [16]
Caucasian	893^1^	44%	493	39%	−^5^	Struch et al. 2008 [15]
Asian (Thai)	54	0%	43	2%	−	Anusaksathien et al. 2003 [18]
Japanese	64^1^	6%	64	10%	−	Soga et al. 2003 [29]
Japanese	58^2^	5%	44	7%	−	Kobayashi et al. 2007 [19]
Japanese	100^2^	6%	100	6%	−	Kobayashi et al. 2007 [20]
Brazilian	52	44%	31	23%	+	Moreira et al. 2005 [30]
Brazilian	29	28%	17	18%	−	Gonçalves et al. 2006 [21]
Brazilian	117	39%	175	31%	−	Ferreira et al. 2008 [31]
Indian	30	30%	31	23%	−	Kaarthikeyan et al. 2009 [32]

nr = not reported. − = association not found. + = association found.

^1^Cases diagnosed as adult periodontitis.

^2^Cases diagnosed as mixed periodontitis status.

^3^An association with periodontitis was found for combined genotype: carriage of *R*-allele for* IL1A *-889, *IL1B* +3954, and *IL1RN* in a subgroup of patients being nonsmokers, and at the same time culture negative for *P. gingivalis *and* A. actinomycetemcomitans.*

^4^ *N*-allele is associated with CP.

^5^ *R*-allele is not quit associated with CP (*P* = .07).

**Table 3:** *IL1B *-511 (-31) and *IL1RN *VNTR (+2018) gene polymorphisms and carriage rate of the *Rare* (*R*)-allele in case-control studies and association with susceptibility to chronic periodontitis.

		Patients		Controls			
*IL1* gene	Ethnicity of subjects	*n*	*R*-allele	*n*	*R*-allele	Associated	Reference
polymorphism			carriage		carriage	with periodontitis	
*B *-511 (-31) C>T	Caucasian	32^1^	59%	32	59%	−	Gore et al. 1998 [5]
	Caucasian	57	53%	100	49%	−	Brett et al. 2005 [10]
	Caucasian	51^1^	43%	168	56%	−	Geismar et al. 2008 [16]
	Japanese	64^1^	67%	64	78%	−	Soga et al. 2003 [29]

*RN *VNTR (+2018 C>T)	Caucasian	105^1^	46%	53	38%	− (+^3^)	Laine et al. 2001 [6]
	Caucasian	51	45%	190	7%	+	Berdeli et al. 2006 [35]
	Caucasian	56	45%	90	30%	−	Sakellari et al. 2006 [12]
	Caucasian	51^1^	34%	168	44%	−	Geismar et al. 2008 [16]
	Japanese	100^2^	6%	100	13%	−	Kobayashi et al. 2007 [20]

nr = not reported. − = association not found. + = association found.

^1^Cases diagnosed as adult periodontitis.

^2^Cases diagnosed as mixed periodontitis status.

^3^An association with periodontitis was found for combined genotype: carriage of *R*-allele for *IL1A *-889, *IL1B* +3954, and *IL1RN* in a subgroup of patients being non-smokers and culture negative for *P. gingivalis *and* A. actinomycetemcomitans.*

**Table 4:** *IL1* composite genotype, that is, *Rare (R*)-allele carriage at *IL1A *-889 (+4845) and *IL1B *+3954 (+3953) [36], in case-control studies and association with susceptibility to chronic periodontitis.

	Patients		Controls			
Ethnicity	*n*	*R*-allele	*n*	*R*-allele	Associated	Reference
of subjects		carriage		carriage	with periodontitis	
Caucasian	32^2^	34%	32	28%	−	Gore et al. 1998 [5]
Caucasian^1^	44	41%	46	28%	+	McDevitt et al. 2000 [37]
Caucasian	105^2^	46%	53	42%	−	Laine et al. 2001 [6]
Caucasian	132^3^	45%	73	42%	−	Papapanou et al. 2001 [38]
Caucasian	61	28%	800	35%	−	Thomson et al. 2001 [7]
Caucasian	84^2^	26%	60	30%	−	Rogers et al. 2002 [8]
Caucasian	402	38%	414	34%	− (+^4^)	Meisel et al. 2003 [39]
Caucasian	45	34%	110	30%	−	Sakellari et al. 2003 [9]
Caucasian	330^3^	26%	101	10%	+	Lopez et al. 2005 [11]
Caucasian	56	41%	90	44%	−	Sakellari et al. 2006 [12]
Chinese	244^2^	0%	56	3%	−	Armitage et al. 2000 [40]
Asian (Thai)	54	0%	43	2%	−	Anusaksathien et al. 2003 [18]
Japanese	100^3^	0.2%	100	0.2%	−	Kobayashi et al. 2007 [20]
Indian	90	14%	30	0%	+	Agrawal et al. 2006 [41]
Brazilian	29	3%	17	12%	−	Gonçalves et al. 2006 [21]

nr = not reported. − = association not found. + = association found.

^1^82% of study population is of Caucasian heritage; results found after multiple logistic regression analysis correcting for smoking status and age.

^2^Cases diagnosed as adult periodontitis.

^3^Cases diagnosed as mixed periodontitis status.

^4^In smokers.

**Table 5:** *TNFA* gene polymorphisms and carriage rate of the *Rare* (*R*)-allele in case-control studies and association with susceptibility to chronic periodontitis.

		Patients		Controls			
*TNFA* gene	Ethnicity	*n*	*R*-allele	*n*	*R*-allele	Associated	Reference
polymorphism	of subjects		carriage		carriage	with periodontitis	
-1031 T>C	Japanese	64^2^	36%	64	22%	+	Soga et al. 2003 [29]

-863 C>A	Japanese	64^2^	39%	64	25%	+	Soga et al. 2003 [29]

-857 C>T	Japanese	64^2^	39%	64	28%	+	Soga et al. 2003 [29]

-367 G>A	Mixed^1^	90	2%	264	2%	−	Craandijk et al. 2002 [42]

-308 G>A	Caucasian	32^2^	28%	32	24%	−	Galbraith et al. 1998 [43]
	Caucasian	40^2^	20%	45	24%	+^3^	Galbraith et al. 1999 [25]
	Caucasian	132	21%	114	24%	−	Fassmann et al. 2003 [44]
	Caucasian	81	36%	80	28%	−	Folwaczny et al. 2004 [45]
	Caucasian	60	22%	39	18%	−	Donati et al. 2005 [46]
	Caucasian	57	35%	100	40%	−	Brett et al. 2005 [10]
	Caucasian	56	16%	90	27%	−	Sakellari et al. 2006 [12]
	Caucasian	51	31%	178	23%	−	Tervonen et al. 2007 [13]
	Caucasian	54	31%	52	35%	−	Schulz et al. 2008 [47]
	Mixed^1^	90	27%	264	29%	−	Craandijk et al. 2002 [42]
	Japanese	64^2^	2%	64	3%	−	Soga et al. 2003 [29]
	Brazilian	74	31%	51	44%	−	De Menezes et al. 2008 [48]

-238 G>A	Caucasian	32^2^	6%	32	6%	−	Galbraith et al. 1998 [43]
	Caucasian	54	9%	52	15%	−	Schulz et al. 2008 [47]
	Mixed^1^	90	6%	264	6%	−	Craandijk et al. 2002 [42]
	Japanese	64^2^	0%	64	3%	−	Soga et al. 2003 [29]

+489 G>A	Mixed^1^	90	24%	264	19%	−	Craandijk et al. 2002 [42]

nr = not reported. − = association not found. + = association found.

^1^81% of study population is of Caucasian heritage.

^2^Cases diagnosed as adult periodontitis.

^3^ *N/N* genotype is associated with CP.

**Table 6:** *IL4 *and* IL4RA *gene polymorphisms and carriage of the *Rare* (*R*)-allele in case-control studies, and association with chronic susceptibility to periodontitis.

		Patients		Controls			
*IL4* gene	Ethnicity of subjects	*n*	*R*-allele	*n*	*R*-allele	Associated	Reference
polymorphism			carriage		carriage	with periodontitis	
-33 C>T	Caucasian	194	32%	158	25%	− (+^2^)	Holla et al. 2008 [49]
	Brazilian	69	68%	44	57%	−	Scarel-Caminaga et al. 2003 [50]
	African-American	30^1^	87%	30	81%	−	Pontes et al. 2004 [51]

-590 C>T	Caucasian	194	32%	158	25%	− (+^2^)	Holla et al. 2008 [49]
	Iranian	26	33%	56	52%	−	Hooshmand et al. 2008 [52]
	African-American	30^1^	67%	30	57%	−	Pontes et al. 2004 [51]

VNTR intron 3	Caucasian	194	31%	158	25%	− (+^2^)	Holla et al. 2008 [49]

*RA *Q551R	Caucasian	60	45%	39	39%	−	Donati et al. 2005 [46]

nr = not reported. − = association not found. + = association found.

^1^Cases diagnosed as mixed periodontitis status.

^2^Haplotype T(-590)/T(-33)/allele 2 (70 bp) is associated with CP (17.0% cp versus 11.0%; OR 1.85).

**Table 7:** *IL6 *and* IL6R *gene polymorphisms and carriage of the *Rare* (*R*)-allele in case-control studies and association with susceptibility to chronic periodontitis.

		Patients		Controls			
*IL6* gene	Ethnicity	*n*	*R*-allele	*n*	*R*-allele	Associated	Reference
polymorphism	of subjects		carriage		carriage	with periodontitis	
-174 G>C	Caucasian	148	77%	107	84%	−	Holla et al. 2004 [57]
	Caucasian	57	61%	100	44%	+	Brett et al. 2005 [10]
	Caucasian	124	42%	116	28%	+^2^	Babel et al. 2006 [58]
	Caucasian	137	65%	82	62%	−	Wohlfahrt et al. 2006 [59]
	Caucasian	51	78%	178	79%	−	Tervonen et al. 2007 [13]
	Caucasian	326	61%	144	71%	+^3^	
	Afro-American	93	10%	45	16%	−	Nibali et al. 2009 [60]
	Asian	87	20%	29	24%	−	
	Brazilian	48	37%	36	67%	+^4^	Trevilatto et al. 2003 [61]
	Japanese	112	0%	77	0%	−	Komatsu et al. 2005 [62]
	Brazilian	155^1^	44%	54	37%	−	Moreira et al. 2007 [63]

-190 C>T	Japanese	112	0%	77	0%	−	Komatsu et al. 2005 [62]

-572 C>G	Caucasian	148	6%	107	20%	+^4^	Holla et al. 2004 [57]
	Japanese	112	37%	77	47%	−	Komatsu et al. 2005 [62]
	Caucasian	326	10%	144	8%	−	
	Afro-American	93	21%	45	13%	−	Nibali et al. 2009 [60]
	Asian	87	61%	29	55%	−	

-373	Japanese	112	12%	77	21%	+^5^	Komatsu et al. 2005 [62]
(A(n)T(m)			(A9T11)		(A9T11)		

-597 G>A	Caucasian	148	78%	107	84%	−	Holla et al. 2004 [57]
	Japanese	112	0%	77	0%	−	Komatsu et al. 2005 [62]

-1363 G>T	Caucasian	326	14%	144	22%	+	
	Afro-American	93	1%	45	4%	−	Nibali et al. 2009 [60]
	Asian	87	5%	29	14%	−	

-1480 C>G	Caucasian	326	58%	144	56%	−	
	Afro-American	93	8%	45	16%	−	Nibali et al. 2009 [60]
	Asian	87	19%	29	24%	−	

-6106 A>T	Caucasian	326	38%	144	37%	−	
	Afro-American	93	36%	45	38%	−	Nibali et al. 2009 [60]
	Asian	87	38%	29	48%	−	

*R* +48892 A>C	Japanese	169	66%	70	66%	− (+^6^)	Galicia et al. 2006 [64]

*R* -183 G>A	Japanese	169	76%	70	74%	−	Galicia et al. 2006 [64]

nr = not reported. − = association not found. + = association found.

^1^Cases diagnosed as mixed periodontitis status.

^2^Only *R/R* genotype frequency is reported and is associated with CP

^3^IL-6 -174, -1363, and -1480 haplotype is associated with periodontitis.

^4^ *N/N* genotype is associated with CP.

^5^Carriage rate of the -373 A9T11 allele higher in non-CP.

^6^ *N*-allele is associated with CP.

**Table 8:** *IL10 *gene polymorphisms and carriage rate of the *Rare* (*R*)-allele in case-control studies and association with susceptibility to chronic periodontitis.

		Patients		Controls			
*IL10* gene	Ethnicity	*n*	*R*-allele	*n*	*R*-allele	Associated	Reference
polymorphism	of subjects		carriage		carriage	with periodontitis	
-1087 (-1082) A>G	Caucasian	60	77%	39	69%	− (+^3^)	Berglundh et al. 2003 [65]
	Caucasian	57	67%	100	69%	−	Brett et al. 2005 [10]
	Caucasian	118	69%	114	74%	−	Babel et al. 2006 [58]
	Caucasian	51	63%	178	70%	−	Tervonen et al. 2007 [13]
	Caucasian	27	81%	34	70%	−	Reichert et al. 2008 [66]
	Mixed^1^	67	49%	43	61%	‒	Scarel-Caminaga et al. 2004 [67]
	(Caucasian)	(48)	(44%)	(36)	(61%)	(−)	

-819 (-824) C>T	Caucasian	27	26%	34	32%	−	Reichert et al. 2008 [66]
	Mixed^1^	67	76%	43	51%	+	Scarel-Caminaga et al. 2004 [67]
	(Caucasian)	(48)	(77%)	(36)	(47%)	(+)	
	Turkish	75	56%	73	45%	−	Sumer et al. 2007 [68]

-627 C>A	Caucasian	57	32%	100	40%	−	Brett et al. 2005 [10]

-592 (-597) C>A	Mixed^1^	67	72%	43	51%	+	Scarel-Caminaga et al. 2004 [67]
	(Caucasian)	(48)	(75%)	(36)	(47%)	(+)	
	Mixed^2^	116	71%	173	51%	+	Claudino et al. 2008 [69]
	Turkish	75	68%	73	41%	+	Sumer et al. 2007 [68]

-590 C>A	Caucasian	27	26%	34	32%	−	Reichert et al. 2008 [66]

nr = not reported. − = association not found. + = association found.

^1^76% of CP and 84% of the control population were Caucasians.

^2^78% of CP and 79% of the control population were Caucasians.

^3^ *N*-allele associated with periodontitis, in particular non-smoking homozygous *N/N* subjects.

**Table 9:** *Fc*γ*RIIa*, *Fc*γ*RIIIa,* and *Fc*γ*RIIIb *gene polymorphisms and carriage rate of the *Rare* (*R*)-allele in case-control studies and association with susceptibility to chronic periodontitis.

		Patients		Controls			
F*c* *γ*R gene	Ethnicity	*n*	*R*-allele	*N*	*R*-allele	Associated	Reference
polymorphism	of subjects		carriage		carriage	with periodontitis	
*IIa* 131 H>R	Caucasian^1^	54	76%	24	71%	−	Colombo et al. 1998 [70]
	Caucasian	56	70%	61	75%	−	Loos et al. 2003 [71]
	Caucasian	213	63%	209	75%	− (+^4^)	Yamamoto et al. 2004 [72]
	Caucasian	132	72%	72	74%	−	Wolf et al. 2006 [73]
	Japanese^2^	100	44%	105	40%	−	Kobayashi et al. 1997 [74]
	Japanese^2^	83	46%	104	39%	−	Kobayashi et al. 2000 [75]
	Japanese	89	42%	64	42%	−	Kobayashi et al. 2001 [76]
	Taiwanese	50	50%	74	62%	−	Chung et al. 2003 [77]
	Japanese	58^3^	48%	44	36%	−	Kobayashi et al. 2007 [19]
	Japanese	100^3^	44%	100	39%	−	Kobayashi et al. 2007 [20]

*IIIa* 158 F>V	Caucasian	56	73%	61	59%	−	Loos et al. 2003 [71]
	Japanese^2^	100	42%	104	46%	−	Sugita et al. 1999 [78]
	Japanese^2^	83	43%	104	46%	−	Kobayashi et al. 2000 [75]
	Japanese	89	49%	64	39%	−	Kobayashi et al. 2001 [76]
	Japanese	58^3^	40%	44	45%	−	Kobayashi et al. 2007 [19]
	Japanese	100^3^	45%	100	45%	−	Kobayashi et al. 2007 [20]

*IIIb NA1> NA2*	Caucasian^1^	54	89%	24	75%	−	Colombo et al. 1998 [70]
	Caucasian	56	88%	61	92%	−	Loos et al. 2003 [71]
	Caucasian	132	84%	72	89%	−	Wolf et al. 2006 [73]
	Japanese^2^	100	63%	105	64%	−	Kobayashi et al. 1997 [74]
	Japanese^2^	83	64%	104	64%	−	Kobayashi et al. 2000 [75]
	Japanese	89	62%	64	56%	−	Kobayashi et al. 2001 [76]
	Japanese^2^	73	74%	46	56%	+	Sugita et al. 2001 [79]
	Japanese^2^	52	58%	55	57%	−	Yoshihara et al. 2001 [80]
	Taiwanese	50	62%	74	55%	−	Chung et al. 2003 [77]
	Japanese	58^3^	66%	44	55%	−	Kobayashi et al. 2007 [19]
	Japanese	100^3^	66%	100	64%	−	Kobayashi et al. 2007 [20]

nr = not reported. − = association not found. + = association found.

^1^Mainly Caucasian, actual % of subjects of non-Caucasian heritage is not provided.

^2^Cases diagnosed as adult periodontitis.

^3^Cases diagnosed as mixed periodontitis status

^4^ *N*-allele is associated with periodontitis in smokers.

**Table 10:** The vitamin D receptor (*VDR*) gene polymorphisms and carriage rate of the *Rare* (*R*)-allele in case-control studies and association with susceptibility to chronic periodontitis.

		Patients		Controls			
*VDR *gene	Ethnicity	*n*	*R*-allele	*n*	*R*-allele	Associated	Reference
polymorphism	of subjects		carriage		carriage	with periodontitis	
*Taq1 *T>C	Caucasian	57	49%	100	78%	+	Brett et al. 2005 [10]
	Caucasian	58	53%	140	63%	+ ^3^	Nibali et al. 2008 [81]
	Chinese^1^	24	4%	39	5%	−	Sun et al. 2002 [82]
	Japanese	74	11%	94	23%	+ ^4^	Tachi et al. 2003 [83]
	Brazilian	69	67%	44	45%	+ (+^5^)	de Brito et al. 2004 [84]
	Turkish	72	50%	102	42%	− (+^6^)	Gunes et al. 2008 [85]

*Bsm1 *A>G	Japanese^1^	52	21%	55	20%	−	Yoshihara et al. 2001 [80]
	Japanese	17	23%	80^2^	19%	− (+^7^)	Naito et al. 2007 [86]
	Brazilian	69	86%	44	82%	− (+^5^)	de Brito Junior et al. 2004 [84]
	Turkish	72	86%	102	91%	− (+^6^)	Gunes et al. 2008 [85]

*Fok1 *A>G	Japanese	74	63%	94	54%	−	Tachi et al. 2003 [83]
	Japanese	17	47%	80^6^	69%	−(+^7^)	Naito et al. 2007 [86]

*Apa1 *G>T	Japanese	17	30%	80^6^	53%	− (+^7^)	Naito et al. 2007 [86]
	Turkish	72	54%	102	61%	− (+^6^)	Gunes et al. 2008 [85]

nr = not reported. − = association not found. + = association found.

^1^Cases diagnosed as adult periodontitis.

^2^Control group consists of patients without severe periodontitis.

^3^The *N/N* genotype is associated with periodontitis in smokers.

^4^The *N*-allele is associated with periodontitis, also when adjusted for smoking and diabetes.

^5^The *Bsm1/Taq1 N/N* haplotype is associated with periodontitis.

^6^The *Apa1/Bsm1/Taq1* haplotype is associated with severe periodontitis.

^7^The *Apa1/Bsm1/Fok1* haplotype is associated with severe periodontitis.

**Table 11:** *CD14, TLR2, *and* TLR4 *gene polymorphisms and carriage rate of the *Rare* (*R*)-allele in case-control studies and association with susceptibility to chronic periodontitis.

		Patients		Controls			
	Ethnicity	*n*	*R*-allele	*n*	*R*-allele	Associated	Reference
	of subjects		carriage		carriage	with periodontitis	
*CD14 *-260^1^ C>T	Caucasian	135	74%	207	70%	−	Holla et al. 2002 [87]
	Caucasians	70	66%	75	76%	− (+^3^)	Folwaczny et al. 2004 [88]
	Caucasian	60	67%	39	77%	+^4^	Donati et al. 2005 [46]
	Caucasian^2^	100	74%	99	71%	+^5^	Laine et al. 2005 [89]
	Caucasian	95	75%	94	77%	−	James et al. 2007 [90]
	Caucasian	51	47%	178	57%	− (+^6^)	Tervonen et al. 2007 [13]
	Caucasian	60	67%	80	64%	−	Schulz et al. 2008 [91]
	Caucasian^2^	72	76%	35	80%	−	Nicu et al. 2009 [92]
	Non-Caucasian^2^	33	64%	22	86%	−	
	Japanese	163	75%	104	82%	− (+^7^)	Yamazaki et al. 2003 [93]

*CD14 *-1359	Caucasian	135	43%	207	42%	−	Holla et al. 2002 [87]
	Caucasian	95	38%	94	35%	−	James et al. 2007 [90]

*TLR2 *Arg677Trp	Caucasian	122	0%	122	0%	−	Folwaczny et al. 2004 [88]
	Caucasian	83	0%	106	0%	−	Berdeli et al. 2007 [94]
	Japanese	97	0%	100	0%	−	Fukusaki et al. 2007 [95]
	Chinese	50	100%	100	100%	−	Zhu et al. 2008 [96]

*TLR2 *Arg753Gln	Caucasian	122	3%	122	4%	−	Folwaczny et al. 2004 [88]
	Caucasian	83	13%	106	13%	−	Berdeli et al. 2007 [94]
	Japanese	97	0%	100	0%	−	Fukusaki et al. 2007 [95]
	Chinese	50	0%	100	6%	−	Zhu et al. 2008 [96]

*TLR2 *-183	Japanese	97	0%	100	1%	−	Fukusaki et al. 2007 [95]

*TLR2 *-148	Japanese	97	0%	100	1%	−	Fukusaki et al. 2007 [95]

*TLR2 *-146	Japanese	97	0%	100	1%	−	Fukusaki et al. 2007 [95]

*TLR2* +1350	Japanese	97	40%	100	28%	−	Fukusaki et al. 2007 [95]

*TLR2* +2343	Japanese	97	0%	100	3%	−	Fukusaki et al. 2007 [95]

*TLR4* Asp299Gly	Caucasian	122	4%	122	3%	−	Folwaczny et al. 2004 [88]
	Caucasian	57	11%	100	7%	−	Brett et al. 2005 [10]
	Caucasian^2^	100	10%	99	9%	−	Laine et al. 2005 [89]
	Caucasian	83	5%	106	6%	−	Berdeli et al. 2007 [94]
	Caucasian	171	14%	218	11%	−	Holla et al. 2007 [97]
	Caucasian	95	19%	94	17%	−	James et al. 2007 [90]
	Caucasian	51	25%	178	20%	−	Tervonen et al. 2007 [13]
	Caucasian	60	13%	80	9%	−	Schulz et al. 2008 [91]
	Japanese	97	0%	100	0%	−	Fukusaki et al. 2007 [95]
	Chinese	50	0%	100	0%	−	Zhu et al. 2008 [96]

*TLR4* Thr399Ile	Caucasian	122	4%	122	4%	−	Folwaczny et al. 2004 [88]
	Caucasian	57	7%	100	18%	−	Brett et al. 2005 [10]
	Caucasian^2^	100	10%	99	9%	−	Laine et al. 2005 [89]
	Caucasian	83	4%	106	5%	−	Berdeli et al. 2007 [94]
	Caucasian	171	14%	218	10%	−	Holla et al. 2007 [97]
	Caucasian	95	22%	94	20%	−	James et al. 2007 [90]
	Caucasian	60	13%	80	9%	−	Schulz et al. 2008 [91]
	Japanese	97	0%	100	0%	−	Fukusaki et al. 2007 [95]
	Chinese	50	0%	100	0%	−	Zhu et al. 2008 [96]

*TLR4* +3528	Japanese	97	0%	100	2%	−	Fukusaki et al. 2007 [95]

*TLR4* +3525	Japanese	97	26%	100	29%	+	Fukusaki et al. 2007 [95]

*TLR4* +4022	Japanese	97	0%	100	1%	−	Fukusaki et al. 2007 [95]

*TLR4* +4529	Japanese	97	2%	100	1%	−	Fukusaki et al. 2007 [95]

nr = not reported. − = association not found. + = association found.

^1^Also refered as -159.

^2^Cases diagnosed as adult periodontitis.

^3^The *N-*allele is associated with periodontitis in women.

^4^The *N-*allele is associated with CP.

^5^The *R/R* genotype is associated with CP also after correcting for age, gender, smoking, and presence *A. actinomytemcomitans* and *P. gingivalis*.

^6^The *R-*allele is associated with disease severity.

^7^The *R-*allele associated with early disease development.

**Table 12:** Miscellaneous candidate genes and the corresponding encoded proteins studied in relation to susceptibility to chronic periodontitis and reported association.

Polymorphism	Coded protein	Reference	Associated
in gene			with periodontitis
*ACE*	Angiotensin-converting enzyme	Holla et al. 2001 [98]	− (+^1^)
*BPI*	Bactericidal/permeability-increasing protein	Glas et al. 2006 [99]	−
*CARD15 (NOD2)*	Caspase recruitment domain-15	Folwaczny et al. 2004 [100]	−
		Laine et al. 2004 [101]	−
*CCR5*	Chemokine receptor-5	Folwaczny et al. 2003 [102]	−
		Wohlfahrt et al. 2006 [59]	−
		Savarrio et al. 2007 [103]	−

*COL1A1*	Type 1 collagen	Sakellari et al. 2006 [12]	−
*COX-2*	Cyclooxygenase-2	Ho et al. 2008 [104]	+
		Xie et al. 2009 [105]	+

*CTLA-4*	Cytotoxic T-lymphocyte antigen-4	Wohlfahrt et al. 2006 [59]	−
*DEFB1*	Human $\underset{˙}{\beta }$ defensin $\tilde{\underset{˙}{\beta }}$1	Wohlfahrt et al. 2006 [59]	−
*eNOS*	Endothelial nitric oxide synthase	Berdeli et al. 2006 [106]	+
*ER2*	Estrogen receptor-2	Zhang et al. 2004 [107]	−
*E-selectin*	E-selectin	Houshmand et al. 2009 [108]	+
*ET1*	Endothelin-1	Holla et al. 2001 [98]	−
*FasL*	Fas ligand	Wohlfahrt et al. 2006 [59]	−
*FBR*	Fibrinogen	Sahingur et al. 2003 [109]	+ ^2^
*Fc* *γ* *RIIb*	Fc*γ* receptor IIb	Yasuda et al. 2003 [110]	+
		Kobayashi et al. 2007 [19]	+

*GSTM1*	Glutathione-S-transferase M1	Concolino et al. 2007 [111]	+

*GSTT1*	Glutathione-S-transferase T1	Concolino et al. 2007 [111]	−

*ICAM-1*	Intercellular adhesion molecule-1	Wohlfahrt et al. 2006 [59]	−

*ICOS*	Inducible costimulator	Wohlfahrt et al. 2006 [59]	−

*IFNG*	Interferon *γ*	Hooshmand et al. 2008 [52]	−
		Reichert et al. 2008 [112]	−

*IFNGR1*	Interferon *γ* receptor-1	Fraser et al. 2003 [113]	− (+^3^)
		Babel et al. 2006 [58]	−

*IL2*	Interleukin-2	Scarel-Caminaga et al. 2002 [114]	−

*IL12*	Interleukin-12	Reichert et al. 2008 [112]	−
*IL12RB2*		Takeuchi-Hatanaka et al. 2008 [115]	−

*IL16*	Interleukin-16	Folwaczny et al. 2005 [116]	−
*IL18*	Interleukin-18	Folwaczny et al. 2005 [117]	−
*IL24 *	Interleukin-24	Savarrio et al. 2007 [103]	−
*Lactoferrin*	Lactoferrin	Wu et al. 2009 [118]	−
*L-selectin*	L-selectin	Houshmand et al. 2009 [108]	−
*LTA*	Lymphotoxin-*α*	Holla et al. 2001 [98]	+
		Fassmann et al. 2003 [44]	− (+^4^)

*MBL*	Mannose binding lectin	Louropoulou et al. 2008 [119]	−
		Tsutsumi et al. 2009 [120]	−

*MMP1*	Matrix metalloproteinase-1	de Souza et al. 2003 [121]	− (+^5^)
		Holla et al. 2004 [122]	−
		Itagaki et al. 2004 [123]	−
		Astolfi et al. 2006 [124]	−
		Cao et al. 2006 [125]	+
		Pirhan et al. 2008 [126]	+
		Ustun et al. 2008 [127]	‒

*MMP2*	Matrix metalloproteinase-1 (gelatinase A)	Holla et al. 2005 [128]	−
		Gurkan et al. 2008 [129]	−

*MMP3*	Matrix metalloproteinase-3	Itagaki et al. 2004 [123]	−
		Astolfi et al. 2006 [124]	+

*MMP9*	Matrix metalloproteinase-9	de Souza et al. 2005 [130]	−
		Holla et al. 2006 [131]	−
		Keles et al. 2006 [132]	+
		Gurkan et al. 2008 [129]	−

*MMP12*	Matrix metalloproteinase-12	Gurkan et al. 2008 [129]	−
*MPO*	Myeloperoxidase	Meisel et al. 2002 [133]	− (+^6^)
*NAT2*	N-acetyltransferase-2	Meisel et al. 2000 [134]	+
		Kocher et al. 2002 [135]	−

*OPG*	Osteoprotegerin	Wohlfahrt et al. 2006 [59]	−
		Wagner et al. 2007 [14]	−
		Baioni et al. 2008 [136]	−
		Park et al. 2008 [137]	− (+^7^)

*OPN*	Osteopontin	Wohlfahrt et al. 2006 [59]	−
*PAI1*	Plasminogen-activator-inhibitor-1	Holla et al. 2002 [138]	+
		Gurkan et al. 2007 [139]	−

*RAGE*	Receptor for advanced glycation end products	Holla et al. 2001 [140]	+
*RANTES*	Regelated on activation, normal T cells expressed and secreted	Savarrio et al. 2007 [103]	−
*S100A8 *	Calprotectin	Li et al. 2007 [141]	+ ^8^
*SFTPD*	Surfactant protein D	Glas et al. 2008 [142]	−
*TGFB1*	Transforming growth factor-*β*_1_	Holla et al. 2002 [143]	−
		de Souza et al. 2003 [144]	−
		Atilla et al. 2006 [145]	+
		Babel et al. 2006 [58]	+ ^9^

*TIMP2*	Tissue inhibitor of matrix metalloproteinase	de Souza et al. 2005 [130]	−
*TNFR2*	Tumor necrosis factor receptor-2	Shimada et al. 2004 [146]	+
*t* *-PA*	Tissue plasminogen-activator	Gurkan et al. 2007 [139]	−

− = association not found. + = association found.

^1^in combination with LTA.

^2^ *R*-allele associated with higher serum fibrinogen.

^3^ *R*-allele in combination with smoking.

^4^ *N*-allele protective in combination with *TNFA*-308.

^5^ *R*-allele associated in non-smokers.

^6^ *R*-allele protective for females.

^7^950T and 1181G haplotype is associated with CP.

^8^ *N*-allele of rs3795391and rs3806232 is associated with CP in Chinese males.

^9^ *R*-allele of codon 25 associated with CP.
